# Secondary Macular Hole Closure With Sub-Tenon Triamcinolone Acetonide

**DOI:** 10.1177/24741264251364009

**Published:** 2025-08-14

**Authors:** Reza Azimi, Amr K. Hassan, Elina Ghafari, Baruch D. Kuppermann, Mitul C. Mehta

**Affiliations:** 1 Department of Ophthalmology and Visual Sciences, Gavin Herbert Eye Institute, School of Medicine, University of California, Irvine, CA, USA

**Keywords:** full-thickness macular hole, sub-Tenon triamcinolone acetonide, secondary macular holes, cystoid macular edema, vitreoretinal surgery, traumatic macular hole

## Abstract

**Purpose:** To evaluate the efficacy and safety of sub-Tenon triamcinolone acetonide in the management of secondary full-thickness macular holes after vitreoretinal surgeries or traumatic injury. **Methods:** This case series includes 3 patients with secondary macular holes. All patients were treated with 40 mg sub-Tenon triamcinolone acetonide injections for macular holes. **Results:** Postinjection, the macular holes closed in 2 months, 2 weeks, and 1 month, respectively. Vision improved in all cases. The third patient experienced a significant increase in intraocular pressure, effectively managed with topical medications. No recurrence was observed at follow-up. **Conclusions:** Sub-Tenon triamcinolone acetonide is a promising therapeutic option for the management of secondary macular holes less than 200 µm in diameter, demonstrating efficacy in promoting hole closure and improving visual outcomes.

## Introduction

Full-thickness macular holes are an anatomic defect that can cause significant central vision loss. They are classified as primary (idiopathic) or secondary. Primary macular holes usually result from traction on the fovea caused by an anomalous posterior vitreous detachment (PVD). Secondary macular holes result from various preceding or concurrent pathologic mechanisms, such as surgery, trauma, or inflammation.^1^

The pathophysiology of full-thickness macular holes is still being studied, with the combined tractional-hydration theory as a leading explanation. This theory suggests that both retinal tissue hydration and tractional forces from the vitreous and the internal limiting membrane play significant roles in the formation and repair of macular holes. The process is generally divided into 3 phases: an initiating anteroposterior and tangential traction phase, a progression or hydration phase, and a closure or dehydration and external limiting membrane repair phase.^2^

Secondary macular holes, which can develop after events such as surgery or trauma, present a unique challenge. They require careful consideration for treatment, often beginning with the traditional approach of pars plana vitrectomy (PPV). Although PPV is typically effective, it is invasive and associated with possible postoperative complications, such as intraocular infection, retinal detachment, visual field loss, and the need for subsequent cataract surgery or face-down positioning postsurgery.^2^

Nonsurgical treatments involving topical therapy have proven effective, including steroids, carbonic anhydrase inhibitors, β blockers, and nonsteroidal anti-inflammatory drugs. These therapies are noninvasive and often present fewer complications compared to traditional surgical options. Topical treatments aim to address the underlying pathophysiology of full-thickness macular holes by promoting cystoid dehydration through the retinal pigment epithelium, which enables reestablishment of the external limiting membrane and allows the hole edges to reappose.^1,2^

Despite various reports demonstrating the success of topical therapies, comprehensive studies evaluating their overall efficacy and safety profiles are limited. Additionally, the clinical characteristics linked to successful macular hole closure, whether through medical or surgical intervention, are not completely understood.^1,2^

In this case series, we present 3 cases of secondary macular holes successfully treated with sub-Tenon triamcinolone acetate after complications from prior vitreoretinal procedures or trauma. We present further evidence supporting medical therapy as a viable alternative treatment for secondary macular holes.

## Methods

We provide a case series of 3 adult male patients with secondary macular holes who were treated with 40 mg sub-Tenon triamcinolone acetonide injections for macular holes. Preinjection and postinjection optical coherence tomography (OCT) images were collected. Consent for publication was obtained from all subjects.

No statistical analysis was done, and the cases’ clinical course is described in detail.

## Results

### Case 1

A 38-year-old man presented with recurrent rhegmatogenous retinal detachment (RRD) with proliferative vitreoretinopathy (PVR) grade C in his left eye and best-corrected visual acuity (BCVA) at hand motion (HM) level. The patient initially underwent PPV and silicone oil injection. After 9 months, the silicone oil was removed and a cataract extraction and intraocular lens (IOL) insertion was performed. OCT conducted during the silicone oil-filled period showed no macular holes.

**Figure 1.**
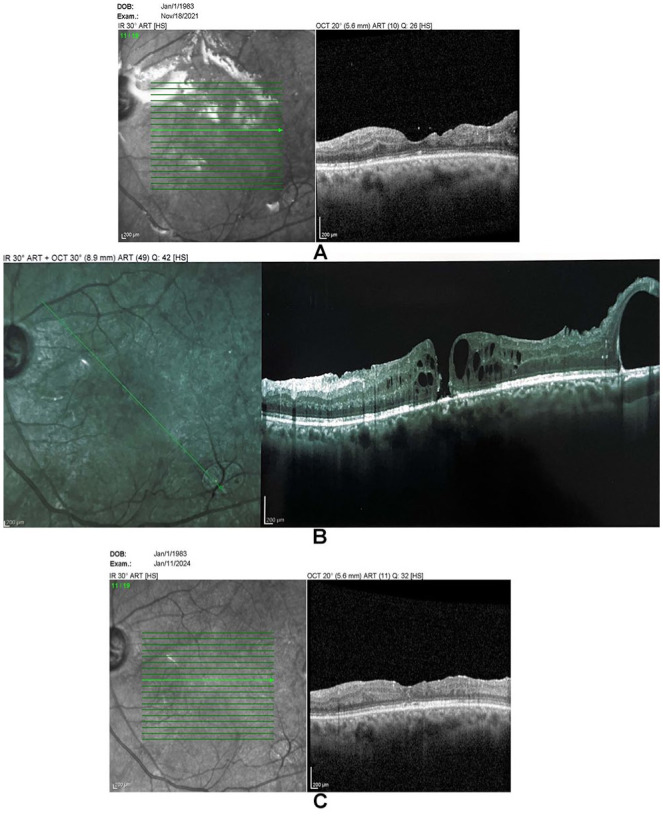
Optical coherence tomography of the macula for case 1 showing (A) post pars plana vitrectomy showing no macular hole under silicone oil, (B) macular hole at 2 months postsurgery, and (C) resolution of the macular hole at 2 months postinjection.

However, follow-up OCT conducted 1 month later revealed cystoid macular edema (CME), and a macular hole developed 1 month afterward. The macular hole diameter was about 100 µm and the BCVA was 20/100. The patient was pseudophakic and received topical steroid (prednisolone) treatment. He received a 40 mg sub-Tenon triamcinolone acetonide injection. Follow-up OCT conducted 2 months later confirmed closure of the macular hole (Figure 1).

The patient had a history of glaucoma in both eyes but did not exhibit significant intraocular pressure elevation postinjection; the intraocular pressure remained controlled with the same medication regimen used before injection. BCVA improved to 20/60. No recurrence was observed at 30-month follow-up.

### Case 2

A 61-year-old pseudophakic man presented with a near-total fresh RRD in his left eye with a superior break. His BCVA was at HM level. PPV with sulfur hexafluoride gas was performed, resulting in total retinal reattachment postsurgery. Follow-up OCT conducted 1 month later revealed a macular hole with a diameter of about 150 µm and a BCVA of counting fingers (CF) at 2 m. Retinal detachment presented from retinal pigment epithelium (RPE) on the temporal side. The patient was receiving topical steroid treatment.

**Figure 2.**
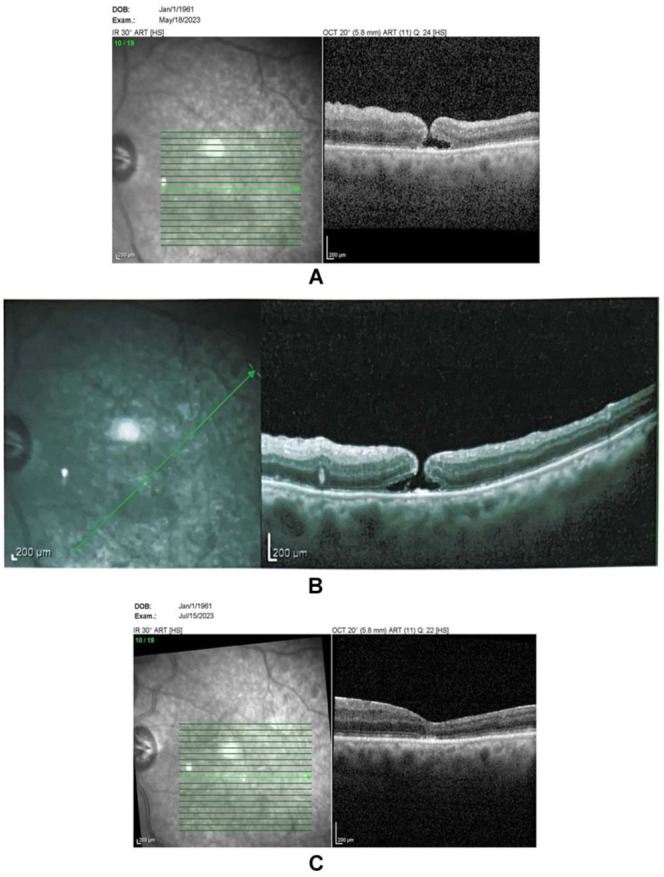
Optical coherence tomography of the macula for case 2 showing (A–B) a macular hole and (C) its resolution 2 months later.

The patient also received a 40 mg sub-Tenon triamcinolone acetonide injection. Follow-up OCTs conducted 2 weeks and 2 months later showed the macular hole had successfully closed (Figure 2). There were no complications related to the sub-Tenon triamcinolone acetonide injection. The patient’s BCVA was CF at 3 m. No recurrence was observed at 18-month follow-up.

### Case 3

**Figure 3.**
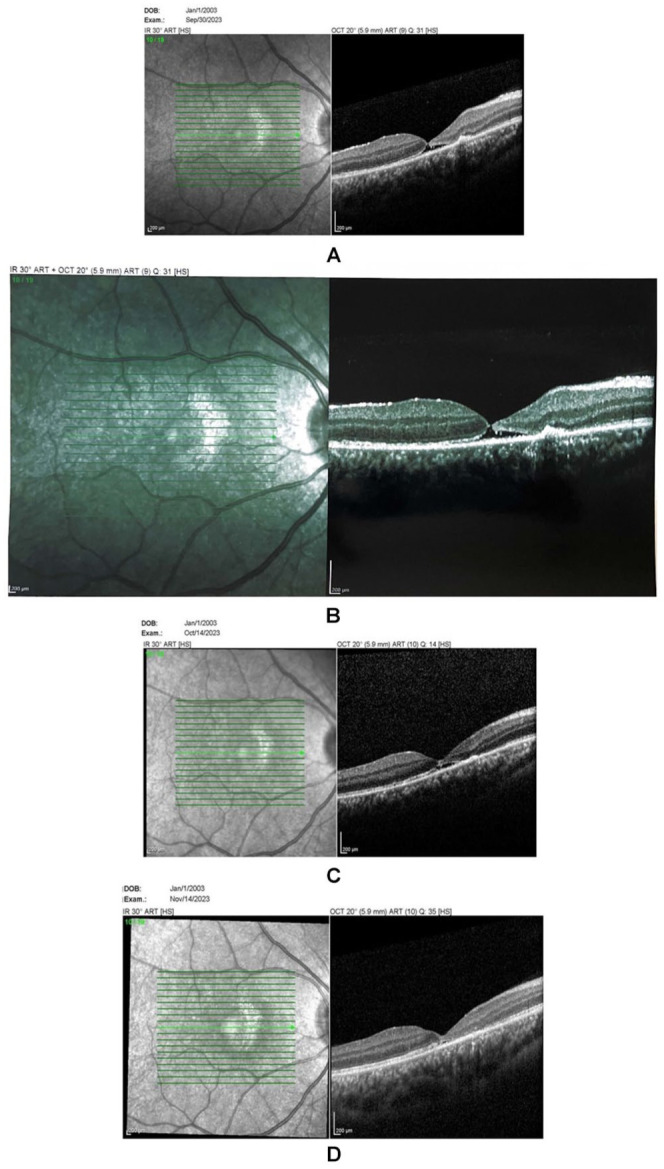
Optical coherence tomography of the macula for case 3 showing (A–B) a macular hole, (C) subretinal fluid 2 weeks after injection, and (D) complete resolution 6 weeks after injection.

A 20-year-old man presented with a traumatic macular hole in his right eye, secondary to a blunt trauma caused by a resistance band. His initial BCVA was 20/100. At 4 weeks post-trauma, the patient underwent cataract extraction and IOL insertion for traumatic cataract. At 4 weeks postsurgery, the patient returned for retinal care, and the OCT confirmed a full-thickness macular hole (Figure 3). The macular hole diameter was about 50 µm, and the BCVA was 20/100. The patient received topical steroid treatment after cataract surgery. He also received a 40 mg sub-Tenon triamcinolone acetonide injection. Follow-up OCTs conducted 2 weeks and 6 weeks later showed successful closure of the macular hole and absorption of subretinal fluid (Figure 3).

The patient experienced a significant rise in intraocular pressure postinjection, effectively controlled with topical medications. After 2 months, the patient gradually weaned off medication. The BCVA improved to 20/50. No recurrence was observed at 12-month follow-up.

Sub-Tenon triamcinolone acetonide injections successfully managed secondary full-thickness macular holes in these cases, highlighting the clinical outcomes and safety profile of this treatment approach (Table 1).

**Table 1.** Cases With Full-Thickness Macular Holes Treated With Sub-Tenon Triamcinolone Acetonide.

Variable	Case 1	Case 2	Case 3
Age (y)	38	61	20
Sex	Male	Male	Male
Diagnosis at presentation	RRD	RRD	Traumatic full-thickness macular hole
Involved eye	Left	Left	Right
Lens status	Pseudophakic	Pseudophakic	Pseudophakic
Steroid use duration	2 mo (1 mo resulting from silicone oil removal and cataract extraction and IOL insertion, and 1 mo resulting from development of CME)	1 mo (resulting from the initial PPV with sulfur hexafluoride gas surgery)	1 mo (resulting from cataract extraction and IOL insertion performed 1 mo after trauma)
BCVA at presentation	HM	HM	20/100
Procedures taken before secondary full-thickness macular hole	PPV with silicone oil; silicone oil removal; cataract extraction and IOL insertion	PPV with sulfur hexafluoride gas; cataract extraction and IOL insertion	Cataract extraction and IOL insertion
BCVA after secondary full-thickness macular hole formation	20/100	CF at 1 m	20/100
Full-thickness macular hole diameter (µm)	100 (<200)	150 (<200)	50 (<200)
BCVA after sub-Tenon triamcinolone acetate	20/60	CF at 2 m	20/50
Complications related to injection	No	No	Transient rise of intraocular pressure
Time to closure from injection	2 mo	2 wk	2 wk
Follow-up time after injection (mo)	30	18	12
Recurrence	No	No	No

Abbreviations: BCVA, best-corrected visual acuity; CF, counting fingers; CME, cystoid macular edema; HM, hand motions; IOL, intraocular lens; PPV, pars plana vitrectomy; RRD, rhegmatogenous retinal detachment.

## Conclusions

Surgical means, such as PPV, successfully manage full-thickness macular holes, often combined with internal limiting membrane (ILM) peeling and gas tamponade. This approach has a high success rate, but it is not without risks. Complications can include intraocular infection, subsequent cataract formation requiring additional surgery, retinal detachment, visual field loss, and the need for patients to maintain a face-down position postoperatively.^2^ Persistent or recurrent macular holes occur in approximately 10%-12% of cases after initial surgical intervention, leading to the exploration of additional reintervention protocols, such as ILM flap transplantation, autologous serum or blood injection, and extensions of ILM peeling.^3^

These complications triggered the search for less invasive treatment of full-thickness macular holes. Sub-Tenon triamcinolone acetonide proved effective in treating persistent macular holes despite PPV and ILM translocation, successfully closing the holes 2 weeks after injection, with closure maintained 1 year after PPV.^4^ Sub-Tenon triamcinolone acetonide achieved success in a different patient with a macular hole resistant to PPV and ILM peeling with an underlying uveitic etiology.^5^ Additionally, a 65-year-old woman with a central retinal vein occlusion (CRVO) who had undergone PPV presented with a persistent macular hole that closed 1 week after injection and remained closed at 3-month follow-up.^6^

Our case series used sub-Tenon triamcinolone acetonide to treat secondary macular holes that developed after surgery or trauma. We chose this approach for its minimal invasiveness and comparatively lower risk of complications. Triamcinolone acetonide offers several advantages, including anti-inflammatory properties that reduce CME and help reappose hole edges.^3^ Additionally, some studies using intravitreal triamcinolone acetonide showed that its mechanical action of plugging the macular defect promoted hole closure.^3^

Previous studies reporting on this treatment strategy focused either on (1) primary macular holes with failed PPV, which differs from our focus on secondary full-thickness macular holes,^4^ or (2) macular holes and CME resulting from a uveitic etiology or CRVO etiologies, where steroid treatment is warranted.^5,6^ Our cases had secondary full-thickness macular holes with no CME, except for the first described case, which highlights the efficacy of sub-Tenon triamcinolone acetonide in treating secondary full-thickness macular holes.

Sub-Tenon triamcinolone acetonide injections can efficiently achieve macular hole closure. Advantages include its minimally invasive nature, reduced surgical risks, anti-inflammatory properties effectively reducing CME, and the mechanical plugging effect aiding anatomical closure.^3^

Traumatic macular holes can undergo spontaneous closure.^7^ However, this is not very common,^8^ occurring in about 37% of cases.^9^ Moreover, after 1 month of observing our patient with traumatic full-thickness macular holes (who was already receiving topical steroid treatment for cataract extraction and intraocular lens (IOL) insertion surgery), we elected to treat with sub-Tenon triamcinolone acetonide. Observation is the standard of care for macular hole cases, and we observed our cases for 1 month each (2 months in case 1) before proceeding with a more aggressive treatment.

Topical treatment is sometimes used in these cases, with a reported success rate of 36.7% for the combination of steroid, carbonic anhydrase inhibitor, and nonsteroidal anti-inflammatory drops.^2^ However, only 14% of the full-thickness macular holes in this study were labeled as secondary.^2^ Another study reported a closure rate of 89% using difluprednate with either a topical carbonic anhydrase inhibitor or a nonsteroidal anti-inflammatory drug.^1^ Our cases underwent steroid therapy for 1 month each (2 months for case 1) before proceeding with sub-Tenon triamcinolone acetonide.

The primary goal of any treatment for macular holes is closing the macular defect. The visual outcome may vary based on several factors, including the size of the macular hole, the duration it has been present, and any preexisting retinal conditions. The cases in this series involved small macular holes (less than 200 µm in diameter), a key factor when considering noninvasive treatment options. Previous studies have indicated that smaller macular holes are more likely to respond to less invasive interventions.^1^ Although our case series demonstrates successful anatomic closure and improvement in visual acuity with sub-Tenon triamcinolone acetonide, achieving the same visual outcomes as traditional surgical methods can be challenging. Previous studies suggest that the final VA post-treatment, whether surgical or nonsurgical, might not differ significantly as the primary goal is closure of the macular hole.^3^

Although edema is a crucial factor in the pathophysiology of macular holes and is often thought to influence treatment response, 2 of the 3 cases presented showed no significant edema. Yet, these cases still responded positively to sub-Tenon triamcinolone acetonide treatment. This observation suggests that mechanisms other than merely reducing edema should be considered when evaluating the efficacy of sub-Tenon steroids. The absence of edema should not deter consideration of sub-Tenon steroid injections as a viable treatment option.

Although some studies have reported recurrences of macular hole with topical therapy, our study showed no recurrence up to the last follow-up.^1,2^ Sub-Tenon triamcinolone acetonide may be more affordable, more convenient, and have a lower recurrence rate compared to topical therapy. However, more research is needed to achieve statistically significant results and comparisons.

This case series supports the potential use of sub-Tenon triamcinolone acetonide as a viable option for the closure of secondary macular holes, especially in cases where the holes are small (<200 µm in diameter) and where surgery poses significant risks. The treatment was generally well-tolerated, with manageable adverse effects. Future studies with larger sample sizes and longer follow-up periods are necessary to substantiate these findings and refine treatment protocols.
